# Effectiveness of 1% versus 0.2% chlorhexidine gels in reducing alveolar osteitis from mandibular third molar surgery: A randomized, double-blind clinical trial

**DOI:** 10.4317/medoral.18702

**Published:** 2013-05-31

**Authors:** Manuel Rodríguez-Pérez, Manuel Bravo-Pérez, José D. Sánchez-López, Esther Muñoz-Soto, María N. Romero-Olid, Pilar Baca-García

**Affiliations:** 1 Department of Stomatology. Faculty of Dentistry. University of Granada, Spain; 2 Oral and Maxillofacial Surgery Department. Virgen de las Nieves University Hospital of Granada, Spain

## Abstract

Purpose: Alveolar osteitis (AO) is the most common postoperative complication of dental extractions. The purpose of this study was to compare the effectiveness of 1% versus 0.2% chlorhexidine (CHX) gel in reducing postoperative AO after surgical extraction of mandibular third molars, and assess the impact of treatment on the Oral HealthRelated Quality of Life (OHRQoL). Material and Methods: This clinical study was a randomized, double-blind clinical trial. Eighty eight patients underwent surgical extraction of one retained mandibular third molar with the intra-alveolar application of 0.2% CHX gel. Afterwards, they were assigned to one of two groups: 1% CHX gel (n=42) or 0.2% CHX gel (n=46). The patients applied the gel twice a day to the wound for one week. All patients were evaluated for AO. Results: In the 0.2% CHX gel group, 13% of AO incidence was found, while in the 1% CHX gel group, AO incidence was 7%, a difference that was not statistically significant. Variables such as sensation of pain and inflammation at baseline and during one week, as well as OHRQoL of the patients at 24 hours and 7 days post-extraction, gave no statistically significant differences. Conclusions: There are no significant differences in AO after surgical extraction of mandibular third molars, when comparing applying 1% CHX gel twice a day for 7 days with 0.2% CHX gel.

** Key words:**Alveolar osteitis, chlorhexidine gel, third molar.

## Introduction

Alveolar osteitis (AO) is the most common postoperative complication of dental extractions (1), affecting the Oral Health Related Quality of Life (OHRQoL) of patients (2). It may also be referred to as alveolitis sicca dolorosa, fibrinolytic alveolitis, or localized osteitis; and Crawford was the first to use the term “dry socket” in 1896. Recently, Blum (3) has suggested the following definition for dry socket: postoperative pain surrounding the alveolus that increases in severity for some period from 1 to 3 days after extraction, followed by partial or total clot loss in the interior of the alveolus, with or without halitosis.

The frequency of AO for all dental extractions ranges from 3% to 4% according to various authors (3). However, the highest incidence, from 5% to 30% (4) of cases, generally occurs following the extraction of retained mandibular third molars.

The exact etiology of AO has not yet been defined. Two basic theories prevail, involving fibrinolytics (5) and bacterial infection (6), while epidemiological studies have identified several risk factors, such as difficulty of extraction, the surgeon’s inexperience, use of oral contraceptives, advanced age, female gender, smoking, immunosuppression, poor oral hygiene, and surgical trauma (7). Accordingly, prevention is held to be the best option (8). Several drugs have been used topically in the prevention of AO, among them antifibrinolytic agents, although antiseptics and antibiotics have proven more successful in prevention to date (4).

The most commonly used antiseptic is chlorhexidine (CHX). A meta-analysis (9) provides clinically significant evidence that a protocol of CHX mouth rinses, beginning on the same day of third molar removal and followed for seven days after extraction, reduces the incidence of AO. A recent review shows that 0.2% CHX gel, applied in the alveolus site twice a day for 7 days post-extraction, may be the best preventive measure (10) because it does not interfere with the local alveolar hemostasis (11).

CHX has been shown to exert greater immediate in vivo antibacterial effect and substantivity than other antiseptics used in the oral cavity (12). The substantivity of CHX is influenced by different factors, among them the concentration (13). Bioadhesive gels with higher concentrations of CHX have shown greater effectiveness in various clinical situations: periodontal treatment (14), after oral surgery procedures (15), implant dentistry (16), plaque control (17), caries prevention (18), and oral wound healing (19). Therefore, the objective of our study was to compare the effectiveness of week-long applications of CHX gel, in concentrations of 0.2% and 1%, in preventing postoperative AO after the extraction of retained mandibular third molars, as well as their impact on the patients OHRQoL.

## Material and Methods

This clinical study was a randomized, clinical trial with two parallel groups, following the Consort statement (20). The study involved the treatment of 88 patients of both genders, 18 to 44 years of age, between January 2009 and January 2011 at the School of Dentistry of the University of Granada, and the Oral and Maxillofacial Surgery Service of Virgen de las Nieves Hospital of Granada (Spain). These patients presented almost one retained mandibular third molar with a difficulty index ranging from 4 through 7 (both included) according to the Koerner scale (21). The degree of difficulty was rated by two pre-calibrated surgeons who also performed the surgery. Exclusion criteria were: taking antibiotics or analgesics in the four days before the procedure, other disease contraindicating oral surgery, AIDS or immunosuppression, pregnancy or lactation in the women, allergy to CHX, articaine, paracetamol or ibuprofen, epinephrine contraindication, the simultaneous extraction of two third molars, any jaw-bone-associated pathology, and uncooperative patients (psychic-motor dysfunction and behavior disorders), or those for whom the extraction took over 30 minutes.

All of the patients in the study gave their written informed consent. The study was approved by the Ethics Committees of the University of Granada and the Virgen de las Nieves Hospital of Granada, and followed the principles of the Helsinki Declaration.

Two pharmaceutical forms of digluconate CHX gel were studied: 0.2% (Laboratorios KIN S.A., Barcelona, Spain) and 1% (GlaxoSmithline Consumer Healthcare, Dublin, Ireland).

Before the surgical treatment, the surgeons registered several variables at the base level, and taught the patients to measure them over a week. Using a millimetric ruler, maximum interincisal aperture (basal, and days 1, 2 and 7), and edema (basal, days 1, 2, 3, 4, 5 and 7) were measured. To determine the latter, the following points were marked: mandibular angle, tragus, lateral canthus, base of nasal wing, labial commisure, and pogonion of the side to be operated. From the mandibular angle to each one of the other points, measurements were made using the ruler. Also noted were the sensation of pain (basal, 3 and 7 hours and daily) and inflammation (basal, 3 and 7 hours, and days 1, 2 and 7) on an analogical visual scale of 0 to 100.

The patients underwent the procedure under local anesthesia (articaine 40mgr/ml- epinephrine 0. 01%; Laboratorios Normon S.A., Madrid, Spain) applied to the inferior alveolar, long buccal and lingual nerves. An enveloped or triangular flap was performed in order to gain access to the third molar, carrying out osteotomy and dental sectioning when necessary. Once the tooth had been extracted, the alveolus was cleaned, the bone edges were smoothed, folicular remnants and granulation tissue were eliminated, and bioadhesive 0.2% chlorhexidine gel was applied inside the alveolus. Finally, the wound was sutured with simple 4/0 silk stitches.

The patients were randomly classified into two groups, 0.2% CHX gel (n= 46) or 1% CHX gel (n=42), by means of a simple allocation using a computer program. All patients were instructed to adhere to topical treatment beginning on the day of intervention and for seven days thereafter using one of the two bioadhesive CHX gels. They were asked to brush their teeth twice a day, using a soft surgical toothbrush, and then apply the corresponding CHX gel on the surgical wound. As postoperative symptomatic treatment, all the patients took 600 mg of ibuprofen every 8 hours and 1 gr paracetamol every 12 hours.

As the main variable we took the appearance (or not) of post-operatory alveolitis during one week, following the diagnostic criteria put forth by Blum (3). Telephone calls were made for follow-up. If the patient complained of pain, an appointment was set for clinical evaluation. The tolerance to treatment was also assessed, reported by the patient using a visual analogical scale of 1 to 5, along with repercussion on the OHRQoL of the patient on days 1 and 7 after surgery. To this end, we used a questionnaire designed by Savin and Ogden (22) consisting of 16 items and five dimensions.

Sample size was estimated following the general rule linked to the standardized difference in a given output variable between the two groups. The estimation was based on detection of a standardized difference of 0.6, which is between moderate (0.5) and large (0.8). This gives a size of 40 patients per group for a power of 75% (?=0.25) and a significance level of ?=0.05. Data analysis was carried out using SPSS Windows 15.0 (SPSS Inc., Chicago, IL). The descriptive and analytical methods applied are indicated in the table and figure.

## Results

A total of 88 patients underwent intervention (88 mandibular third molars). All 46 patients in the 0.2% CHX gel group and 42 patients in the 1% CHX gel group completed the study. The mean age was 26 (range 18 to 44). Forty-six patients were female and 42 were male. Twenty-two patients were smokers, and nine women were taking oral contraceptives.

The risk factors of both groups with regard to sociodemographic variables, clinical variables, and the surgical procedure are shown in table 1. No significant statistical differences were found between the two groups (13% of AO incidence in the 0.2% gel CHX group, 7% in the 1% gel CHX group; ( Table 1). Furthermore, the surgeon did not significantly (with a logistic regression model) influence the group effect (results not shown).

**Table 1:**
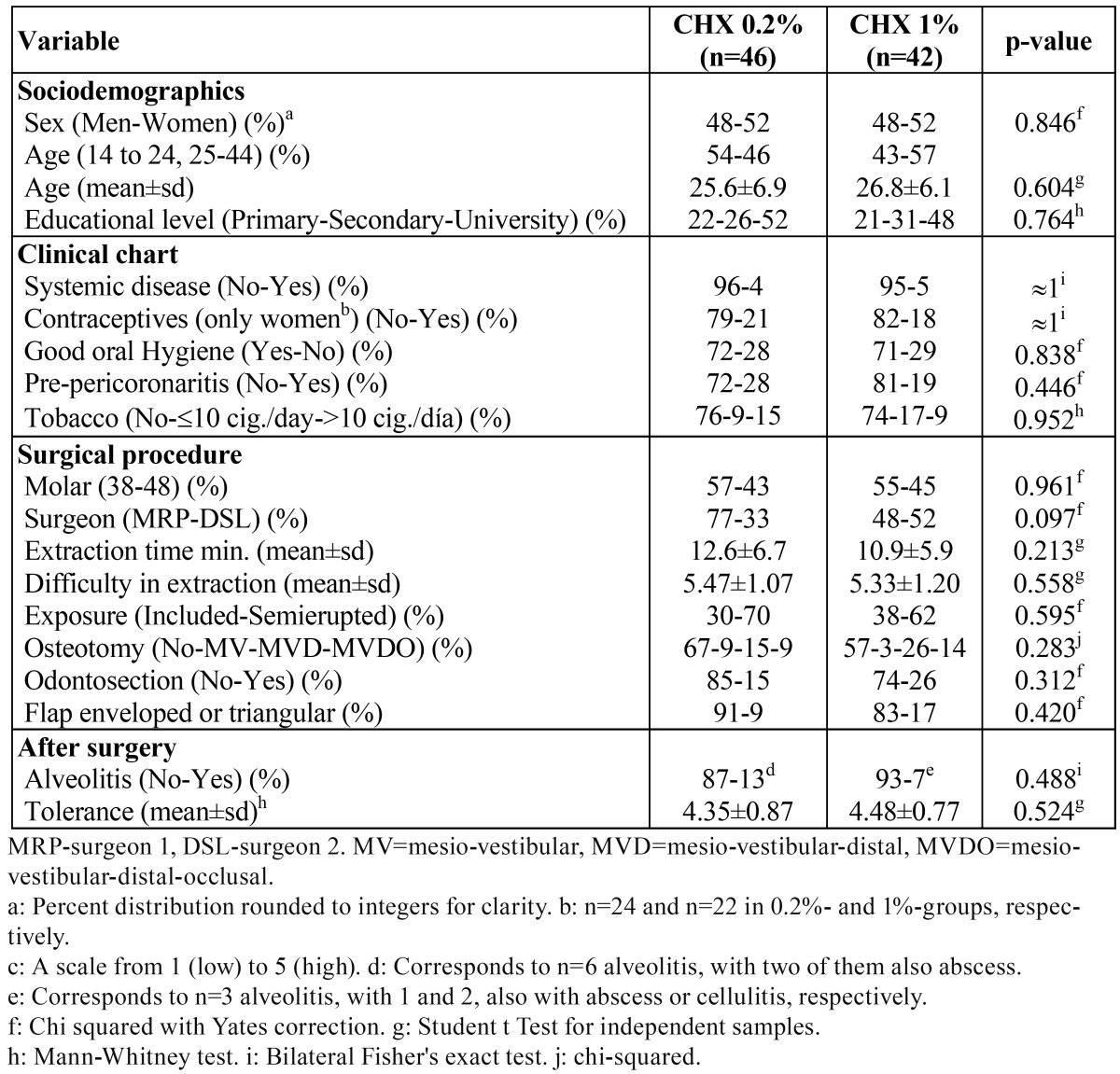
Description and comparison of patients (n=88).

**Figure 1:**
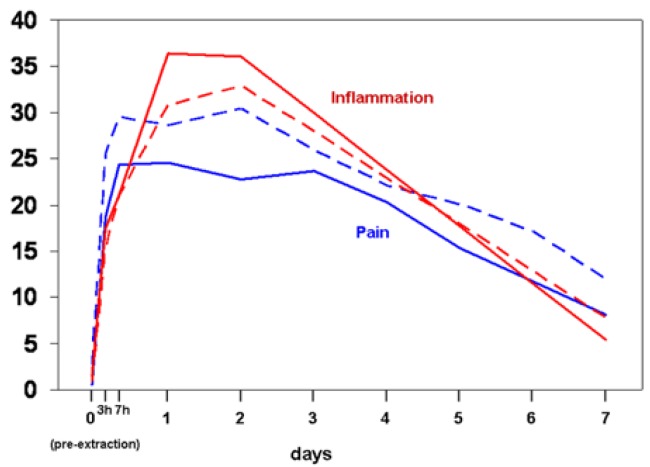
Pain and inflammation, from pre-extraction (day 0) to day 7. Continuous lines refer to group CHX 1%, and discontinuous lines to group CHX 0.2%. Inflammation for days 3 to 6 are extrapolations (not collected). Comparisons between the two groups are non-significant (p>0.05, Mann-Whitney test) for all variables and for all times. All measurements within each line are significantly higher than basal measurements (p<0.05, Wilcoxon’s test).

**Figure 2:**
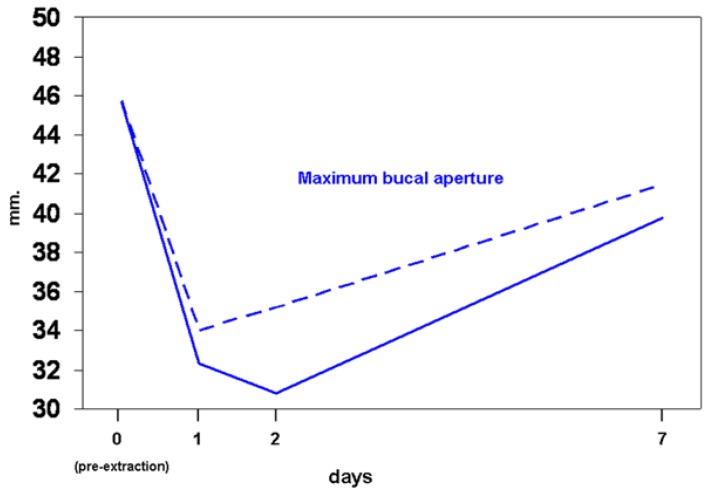
Maximum bucal aperture at pre-extraction (day 0), days 1, 2 and 7. Continuous lines refer to group CHX 1%, and discontinuous lines to group CHX 0.2%. Comparisons between the two groups are non-significant (p>0.05, Mann-Whitney test) for all variables and for all times. All measurements within each line are significantly lower than basal measurements (p<0.05, Wilcoxon’s test).

**Figure 3:**
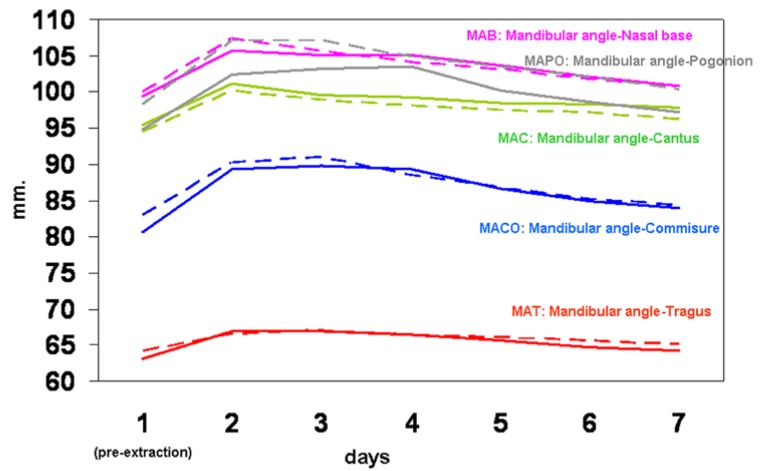
Different facial measurements from mandibular angle, from pre-extraction (day 1) to day 7. Continuous lines refer to group CHX 1%, and discontinuous lines to group CHX 0.2%. Comparisons between both groups are non-significant (p>0.05, Mann-Whitney test) for all variables and for all times. All measurements within each line are significantly higher than basal measurements (p<0.05, Wilcoxon’s test), except for MAB at day 7 (p=0.085).

The baseline subjective sensation of pain, inflammation, and evolution over the week-long follow up, reflected in figure 1, shows no significant differences between the two groups. Figure 2 indicates the maximum interincisal aperture before extraction and at days 1, 2 and 7, with no statistically significant differences seen between the two groups. The baseline level and the evolution of edema are presented in figure 3, again without significant differences for any of the variables studied.

None of the patients displayed adverse effects to the treatment. The results in terms of OHRQoL of the patients at 24 hours and at 7 days post-extraction are given in  Table 2. No statistically significant differences could be established for the two groups for any of the items on day 1 or on day 7. A comparison of the evolution within each group during this week showed improvement in all the dimensions of the questionnaire except for “psychosocial effects”.

**Table 2:**
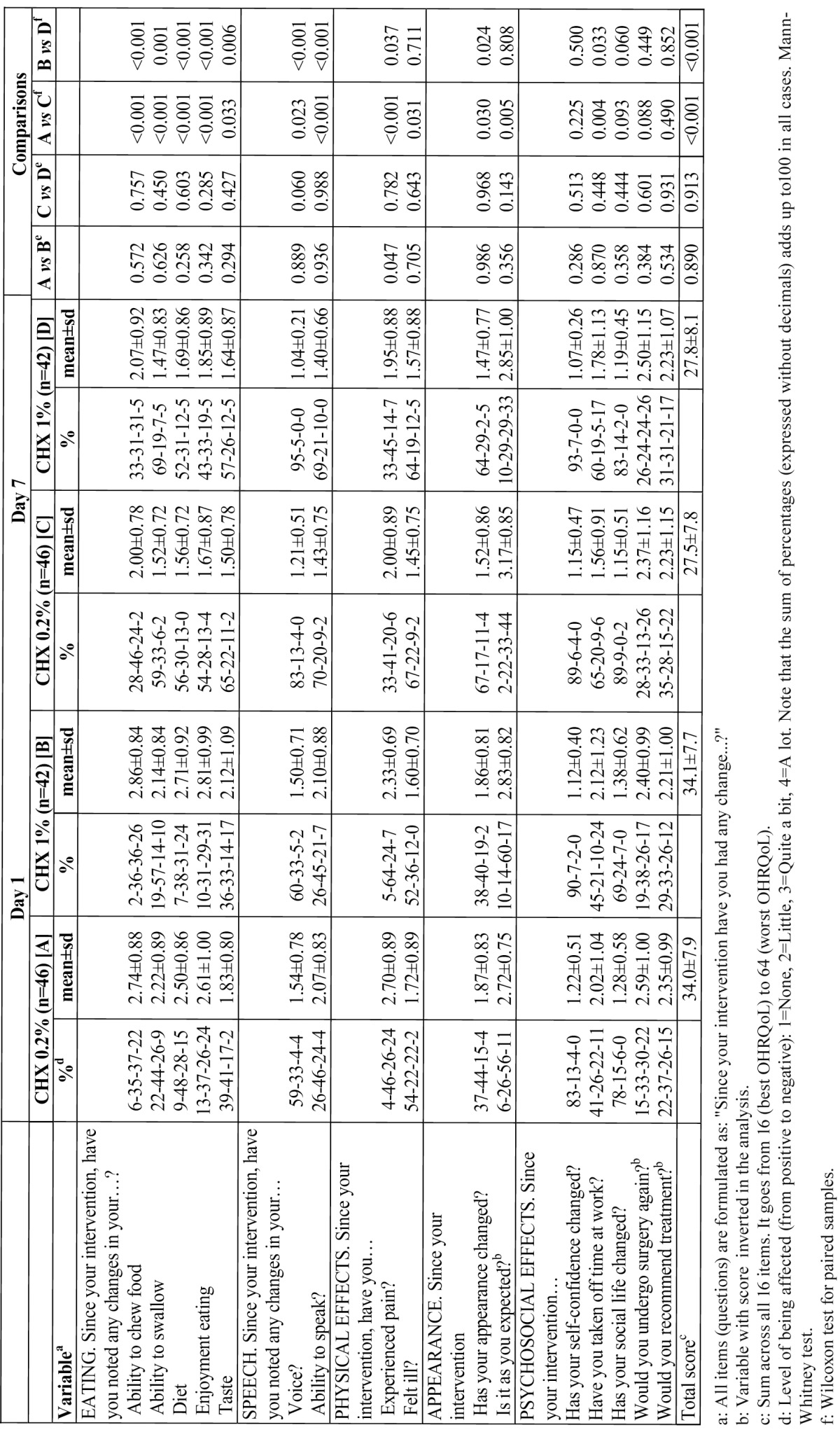
OHRQoL of patients, at days 1 and 7 after surgery (n=88).

## Discussion

In view of the high frequency of AO after surgical extractions, and especially in retained lower third molars (4), and the clinical manifestations that can affect the OHRQoL (23), the prevention of this pathology is particularly relevant. Although the exact etiology of AO has not yet been defined (24), the influence of bacteria appears to be important (5). A number of local and sys-temic risk factors have also been described (4) and should be taken into account in any research about the treatment or prevention of AO.

This study is a double-blind randomized clinical trial (RCTs), following Consort statements (20), a gold standard for studying preventive and therapeutic interventions. The double-blindedness was maintained throughout the study, and comparison of the risk factors derived from the patient and those of the interventions showed there were no significant differences between the two groups established. Any selection bias is therefore unlikely. One possible limitation to be addressed, however, is that our study did not include a control group without treatment. This decision was made when designing the study given the demonstrated efficacy of CHX in the prevention of AO (4,10).

The diagnostic criterion for AO was described by Blum (3) as the one most used in epidemiological studies (10), thus allowing for the comparison of results. The questionnaire about OHRQoL had been previously applied to studies of third molar surgery (22,23), and it is easy for patients to understand and fill out.

Many researchers (4,9,10) have sought to prevent or reduce the incidence of AO. Local treatment with antibiotics, specifically tetracycline, is supported by strong evidence from available RCTs to have a clinically relevant effect on prevention of AO (4), but caution should be taken due to potentially serious adverse effects with its intra-alveolar use, such as hypersensitivity reactions and potential for systemic toxicity (24). The use of CHX is associated with fewer adverse effects (25).

With a high level of evidence, Caso et al. (9) published a meta-analysis of CHX. Its use intra-operatively and 7 days postoperatively appears to reduce the frequency of AO following surgical removal of lower third molars, and in addition, entails no serious adverse effects. However, this meta-analysis only studied CHX in solution. A study evaluating the effects of CHX gel reports that one application of 0.2% CHX bioadhesive gel post-extraction in the alveolus site (15) reduced the incidence of AO by 19% (a significant difference) with respect to a control group undergoing no local treatment. Taking into account this result, it was decided, for ethical reasons, that both groups should receive 0.2% CHX bioadhesive gel post-extraction in the alveolus site. We used CHX gel applied every 12 hours for 7 days after extraction. Although this option is more expensive than CHX in solution, a recent systematic review including studies with CHX gel concludes that it is the best available option for the prevention of AO (10).

Our aim was to improve, if possible, the good results obtained using 0.2% CHX gel (26). The effect of CHX is dose-dependent. A major concentration can increase substantivity (13) and its bactericidal effect (27). Also, the properties of bioadhesive CHX gel, particularly its high viscosity, can reduce the clearance of the active agent from the place of the extractions (28). On the other hand, the use of 1% CHX gel has shown better results in different clinical situations (14-18). We therefore surmised that increasing 5 times the concentration of a bioadhesive CHX gel could further reduce AO incidence. Although the incidence of AO in the 1% CHX gel group was nearly half that obtained for 0.2% CHX gel (7.1% versus 13%), the difference was not statistically significant. These results can be considered in the same interval as those obtained by Hita et al. (26), who used the same methodology, and found 7.5% of AO in their 0.2% gel CHX group as opposed to 25% in a group using 0.12% CHX mouth rinse.

A lack of differences between the two groups of our study was also reflected by the subjective and objective variables studied: evolution of pain, level of inflammation, and interincisal aperture. Assessment of the patients´ OHRQoL one and seven days after surgery showed similar results for the two groups, indicating no substantial difference between using a 1% CHX gel or a 0.2% CHX gel.

Our results could be explained by the use of 0.2% CHX gel inside the alveolus in both groups. Because this procedure in itself has shown an important reduction of AO (19%) (15), achieving a lower incidence was a major challenge. It was not possible using 1% CHX gel inside the alveolus, as we had originally considered, due to possible side effects not yet studied (29). The application of the lower concentration of CHX (0.2%) may result in the formation of a relatively stable monolayer of retained CHX in the oral mucosa, while the higher concentration might have given only an oversaturation of CHX with a rapid release of its excess (30).
